# Epidermal growth factor regulates autophagy activity and endocytosis of yak cumulus cells in a concentration-dependent manner

**DOI:** 10.3389/fvets.2022.1081643

**Published:** 2023-01-09

**Authors:** Rui Ma, Sijiu Yu, Yan Cui, Yangyang Pan, Meng Wang, Libin Wang, Jinglei Wang, Ling Zhao, Hui Zhang

**Affiliations:** ^1^College of Veterinary Medicine, Gansu Agricultural University, Lanzhou, Gansu, China; ^2^Gansu Province Livestock Embryo Engineering Research Center, Lanzhou, Gansu, China

**Keywords:** EGF, autophagy, endocytosis, cumulus cells, yak

## Abstract

**Introduction:**

Autophagy and endocytosis are crucial biological activities in mammalian follicle development and oocyte maturation, which are easily affected by external environmental factors. Epidermal growth factor (EGF), as an important component of follicular fluid, regulates the growth and apoptosis of follicular cells. However, its regulatory mechanism of autophagy and endocytosis in mammals, especially in large domestic animals such as plateau yak, remains unclear. This study aimed to investigate the regulatory mechanism of EGF on autophagy and endocytosis in yak cumulus cells.

**Methods:**

Yak cumulus cells were treated with different concentrations of EGF and appropriate concentrations of EGFR inhibitor gefitinib (10 μM). The dynamic expression levels of Atg5, Beclin1, LC3, Cav1 and Cav2 were detected by immunofluorescence staining, qRT-PCR and Western-blot.

**Results:**

EGF inhibited autophagy in yak cumulus cells by down-regulating the expression of Atg5, Beclin1, and LC3. The level of autophagy varied with the concentration of ligands, and the inhibition was most significant at 100 ng/mL. Noteworthy, EGF can promote endocytosis by regulating the expression of Cav1 and Cav2, but the EGFR-mediated signaling pathway is not the main way to regulate the expression of these proteins.

**Discussion:**

These results provide a reference for further exploring the effects of growth factors on livestock germ cells and the regulatory role of autophagy-endocytosis crosstalk mechanism in follicle development and oocyte maturation, to improve the fecundity of yaks.

## 1. Introduction

Autophagy and caveolin-mediated endocytosis are important physiological regulatory processes of cells, which play a regulatory role in maintaining cell homeostasis, material exchange, and signal transduction (1, 2), and perform an essential function in the regulation of animal reproduction. Studies have shown that autophagy is a pro-survival adaptive response of the bovine cumulus-oocyte complex to heat shock (3). In addition, autophagy regulation not only helps the stress of cumulus cells but also is related to progesterone synthesis and oocyte maturation capacity (4). For instance, studies have found that autophagy leads to an abnormal reduction of porcine oocyte maturation capacity (5). In recent years, endocytosis, as an important way for cells to take in external nutrients, has drawn the attention of researchers. Especially in some relatively new studies, it has been found that the autophagy-endocytosis crosstalk mechanism, as a new cellular regulation mode, is involved in regulating multiple biological processes (6). Studies have shown that endocytosis proteins can be involved in the occurrence of inflammation, cancer, and other diseases by regulating the autophagy activity of cells (7, 8). Endocytosis protein-mediated autophagy regulates LDL endocytosis stimulated by high glucose (9). Noteworthy, the endocytosis protein Cav1 activates autophagy in response to cellular oxidative stress through interaction with Beclin1 (10). In a word, autophagy and endocytosis proteins constitute complex signaling pathways that can regulate multiple biological activities of cells. However, there is a lack of research on follicle development and oocyte maturation in animals.

When stimulated by external factors, cells usually undergo autophagy induction, and the formed autophagic vesicles fuse with lysosomes for digestion and remove components that are not conducive to cell growth (11). During autophagy induction, Beclin1 can form the Beclin1/PI3K complex with 3-phosphatidylinositol kinase (PI3K) to recruit Autophagy-related gene (Atg) 8 and promote the formation of autophagosome membranes (12, 13). Atg5 binds to the Atg12 protein, then Atg5–Atg12, and Atg16 proteins are connected to form an Atg5–Atg12–Atg16 L complex, which can promote the early formation of autophagosomes (14, 15). Microtubule-associated protein 1 light chain 3 (LC3) can be targeted to the autophagosome membrane for promoting the formation of autophagosomes and has a significant regulatory effect on the completion of cellular autophagy (16). The method of endocytosis, which depends on the caveolae, is one of the primary modes for cells to ingest macromolecules from the outside world (17). Caveolin1 (Cav1) and Caveolin2 (Cav2) proteins are anchored in the caveolae to promote the formation of independent vesicles and help cells complete material transportation to take in external nutrients (17, 18). Based on the above theories, the detection of Atg5, Beclin1, LC3, Cav1, and Cav2 expression can be used as an essential indicator of cellular autophagy and endocytosis regulation.

EGF plays a critical role in regulating cell growth by combining with EGFR. Related studies have shown that EGF promotes not only the maturation of oocytes but also their fertilization ability and embryo development and inhibits the apoptosis of oocytes during maturation and promotes the expansion of cumulus cells (19, 20). Notably, the stimulative effect is related to the dose of EGF (21). Because the EGF signaling system of cumulus-oocyte complexes (COCs) matured *in vitro* is not perfect, it is difficult to simulate the physiological environment of follicles, resulting in poor development ability of oocytes *in vitro* and limiting the utilization efficiency of oocytes (22, 23). Therefore, EGF often functions as a helpful additive in cell culture fluids and embryo culture fluids and regulates cell growth through multiple signaling pathways. Current studies have found that EGF has certain effects on the autophagy and endocytosis of mouse cumulus cells (24), but the regulation of EGF on the autophagy and endocytosis of large livestock cumulus cells has not been reported. Gefitinib is an effective EGFR tyrosine kinase inhibitor, effectively inhibiting EGF signaling after acting on cells (25). In the previous work of our laboratory, We have proved that the appropriate concentration of gefitinib can effectively block the cell EGF pathway without affecting the activity of yak cumulus cells, so gefitinib was selected as the EGFR inhibitor in this experiment (26, 27).

Yaks (*Bos grunniens*) have been living in the Qinghai-Tibet Plateau area with high cold and low oxygen for generations. The harsh environment has a particularly great impact on its reproductive performance, so its reproductive capacity is low compared with other cattle breeds (28). Cumulus cells (CCs), as important follicle somatic cells, are tightly wrapped around oocytes. They can exchange substances with oocytes through gap junctions and other ways, and form a signal network through complex paracrine and autocrine factors to achieve bidirectional communication. Follicle development, oocyte maturation, fertilization, and early embryonic development in animals are closely related to the growth state of cumulus cells (29, 30). One study has shown that EGF can affect the apoptosis of yak cumulus cells (31), but its effect on autophagy and endocytosis of yak cumulus cells remains unclear. This study aimed to investigate the regulatory mechanism of EGF involved in autophagy and endocytosis in yak cumulus cells, to lay a reliable foundation for future studies on the mechanism of growth factors on yak oocyte maturation and embryo development.

## 2. Materials and methods

All chemicals used were purchased from Sigma-Aldrich (St. Louis, MO, USA) unless specified otherwise.

### 2.1. Experimental sample collection

The experimental animals were approved by the Animal Ethics Committee of Gansu Agricultural University and treated according to the Animal Ethics Procedures and Guidelines of the People's Republic of China.

About 200 pairs of ovaries of post-pubertal yaks were collected from Xining slaughterhouse in Qinghai Province. The sampling period was September to November, when yaks were in estrus. Ovaries with a volume of 3–8 cm^3^ and a maximum follicle diameter of more than 5 mm were selected. After the yaks are slaughtered, the ovaries shall be collected immediately, put into sterile saline at 32–35°C, and arrived to the laboratory within 4 h.

### 2.2. Isolation and culture of yak cumulus cells

The collected ovaries were washed 3 times in physiological saline pre-equilibrated for 2 h at 37°C. Then used a needle (18–21 gauge) was to extract the fluid from follicles about 10 mm diameter on the surface of the ovary. Injected the follicle fluid into a 15 mL sterile centrifuge tube and placed it at 37°C for 30 min. After natural sedimentation of the COCs, discarded the supernatant liquid, was selected the homogeneous COCs, which wrapped with 3 layers of cumulus cells under a dissecting microscope, shaking vigorously, and collected the scattered CCs. Next, centrifuged at 1,000 rpm/min for 5 min and discarded the supernatant. After centrifugal washing with the culture medium twice, cells were resuspended and cultured in 25 cm^2^ cell culture flasks with DMEM/F12 containing 10% FBS (Fetal Bovine Serum, Qualified, Australia) medium and antibiotics (100 U/mL penicillin, 100 μg/mL streptomycin) at 37°C in a humidified atmosphere of 5% CO_2_.

### 2.3. Immunofluorescent detection of Atg5, Beclin1, LC3, Cav1, and Cav2 proteins in cultured yak cumulus cells (CCs)

After the cell growth was stable, the second generation of yak CCs were fixed in 4% paraformaldehyde at room temperature for 1 h and then permeabilized the cell membrane with 0.5% Triton X-100 for 1 h. Next, 1% BSA was mixed in the cell for 2 h. And cells were separately incubated with anti-Atg5 (1:500 dilution; ab108327, NOVUS, Littleton, US), anti-Beclin1 (1:500 dilution; ab108327, NOVUS, Littleton, US), anti-LC3 (1:200 dilution; ab51520, NOVUS, Littleton, US), anti-Cav1 (1:500 dilution; ab51520, NOVUS, Littleton, US), and anti-Cav2 (1:500 dilution; ab51520, LSbio, Cambridge, US), and anti-β-tubulin (1:250 dilution; bs-0210R, Bioss, Beijing, China) overnight at 4°C. Subsequently, cells were paired with secondary antibody Alexa Fluor 594 labeled Goat anti-rabbit IgG (1:1,000 diluted; Bioss, Beijing, China) and Alexa Fluor 488 labeled Goat anti-rabbit IgG (1:1,000 diluted; Bioss, Beijing, China) incubated for 1 h. We washed each step with PBS 3 times before restaining with DAPI (4'−6-diamidine-2-phenylindole) for 3 min. In the staining results, the red fluorescent label is the target protein, and the blue fluorescent label is the cell nucleus. In this test, PBS was used to replace the primary antibody for incubation as a negative control, and the negative results did not show red fluorescence. The cytoskeletal protein β-tubulin was used as a positive control. The cells were examined with a fluorescence microscope and photographed.

### 2.4. Treatment of CCs with different concentrations of EGF and Gefitinib

The second-generation yak cumulus cells were selected. The cells were divided into 6 groups, and the culture medium of the 6 groups was supplemented with 0, 50, 100, 150, and 200 ng/ml EGF and 10 μM gefitinib, respectively. The serum concentration of the culture medium was 10%. The incubator was set at 37°C with 5% CO_2_ and 21% O_2_. Used the same method to treat the CCs of different groups of yak (set three repeat groups in total). All cells were placed in an incubator for 48 h and then treated for subsequent experiments.

### 2.5. RNA extraction and Real-time quantitative polymerase chain reaction (qRT-PCR)

We used a total RNA kit (OMEGA, Chicago, CA) to extract the RNA of yak cumulus cells and a Go Script reverse transcription system (Promega, Madison, WI, US) to synthesize complementary DNA (cDNA). Primer information of mRNA is shown in Table 1. Real-time polymerase chain reaction (qRT-PCR) (ABIViiA™7; Applied Biosystems, Foster City, CA, US) quantified mRNA levels. The qRT-PCR reaction system consisted of cDNA (2 μL), primer (10 pmol/mL, 0.8 μL), SYBR Premix Ex Taq (II) (10 μL), ROX reference dye II (0.4 μL), and ddH2O (6 μL). The qRT-PCR conditions were 95°C for 10 min (Predenaturation), then 95°C for 15 s (DNA denaturation), 60°C for 30 s (annealing), 72°C for 15 s (extension), then 72°C for 10 min (extension), with 35 cycles. Gene expression levels in each sample were quantified in four replicates using β*-actin* as a reference gene. Used the 2^−ΔΔCt^ method to analyze expression levels of Atg5, Beclin1, LC3, Cav1, and Cav2.

**Table 1:** Primer sequences and cycling conditions used in qRT-PCR.

**Gene**	**Primer sequence (5′ → 3′)**	**Tm/°C**	**Fragment size (bp)**	**Reference sequence**
Cav1	F:GACCCCAAGCATCTCAACGA	56	106	NM_174004.3
	R:TGAAGCTGGCCTTCCAGATG			
Cav2	F:CTGCCTAATGGTCCTGCCTT	58	140	BC134620.1
	R:GGGGCCCAAGTATTCAATCGT			
Atg5	F:AGTTGCTCCTGAAGATGGGG	60	147	NM_001034579.2
	R:TCTGTTGGTTGCGGGATGAT			
Beclin1	F:GAAACCAGGAGAGACCCAGG	56	114	NM_001033627.2
	R:GTGGACATCATCCTGGCTGG			
LC3	R:CCGACTTATCCGAGAGCAGC	59	161	NM_001001169.1
	R:TGAGCTGTAAGCGCCTTCTT			
*β-actin*	F:CGTCCGTGACATCAAGGAGAAGC	60	143	DQ838049.1
	R:GGAACCGCTCATTGCCGATGG			

### 2.6. Western blot

The yak cumulus cells were cleaned 3 times with PBS, and the protein lysis buffer was added to fully lysis the cells for 2 h. We collected the supernatant and stored it in the refrigerator at −80°C. Added sample loading buffer of sodium dodecyl sulfate (SDS) and took a metal bath at 100°C for 15 min to complete protein denaturation. Sodium dodecyl sulfate-polyacrylamide gel electrophoresis (SDS-PAGE) was performed to obtain the target protein. Proteins were transferred to the PVDF membrane by wet transfer. Blocked protein with 5% nonfat milk powder with 0.05% Tween-20 at room temperature for 2 h. Then anti-Atg5 (1:500 dilution; ab108327, NOVUS, Littleton, US), anti-Beclin1 (1:500 dilution; ab108327, NOVUS, Littleton, US), anti-LC3 (1:200 dilution; ab51520, NOVUS, Littleton, US), anti-Cav1 (1:500 dilution; ab51520, NOVUS, Littleton, US), and anti-Cav2 (1:500 dilution; ab51520, LSbio, Cambridge, US) in different groups as the primary antibody incubated membranes at 4°C overnight. The second antibody was incubated at room temperature for 1 h and cleaned with PBST. The electrochemiluminescence (ECL) kit was used for exposure for 1–5 min, and the protein bands were photographed for analysis by Amersham Imager 600 (General Electric Company, USA). IOD values of protein bands were measured by Image-Pro Plus software (protein expression levels = target IOD/ internal reference IOD).

### 2.7. Data analysis

Multiple comparisons were performed using ANOVA and Duncan test. SPSS 21.0 (SPSS Inc., Chicago, IL, USA) was used for statistical analysis. Each group was repeated at least 3 times. Graphpad prism 8.0 is used for preparation results. All data were expressed as mean ±SD, and *P* < 0.05 was considered significant, while values of *P* < 0.01 were considered extremely significant.

### 2.8. Ethics statement

The study was approved by the Animal Ethics Committee of Gansu Agricultural University (Ethic approval file No. GSAU-Eth-VMC-2022-23). All experiments were performed in accordance with the relevant guidelines and regulations.

## 3. Results

### 3.1. Yak cumulus cells culture and distribution of Atg5, Beclin1, LC3, Cav1, Cav2 protein in CCs

**Figure 1:**
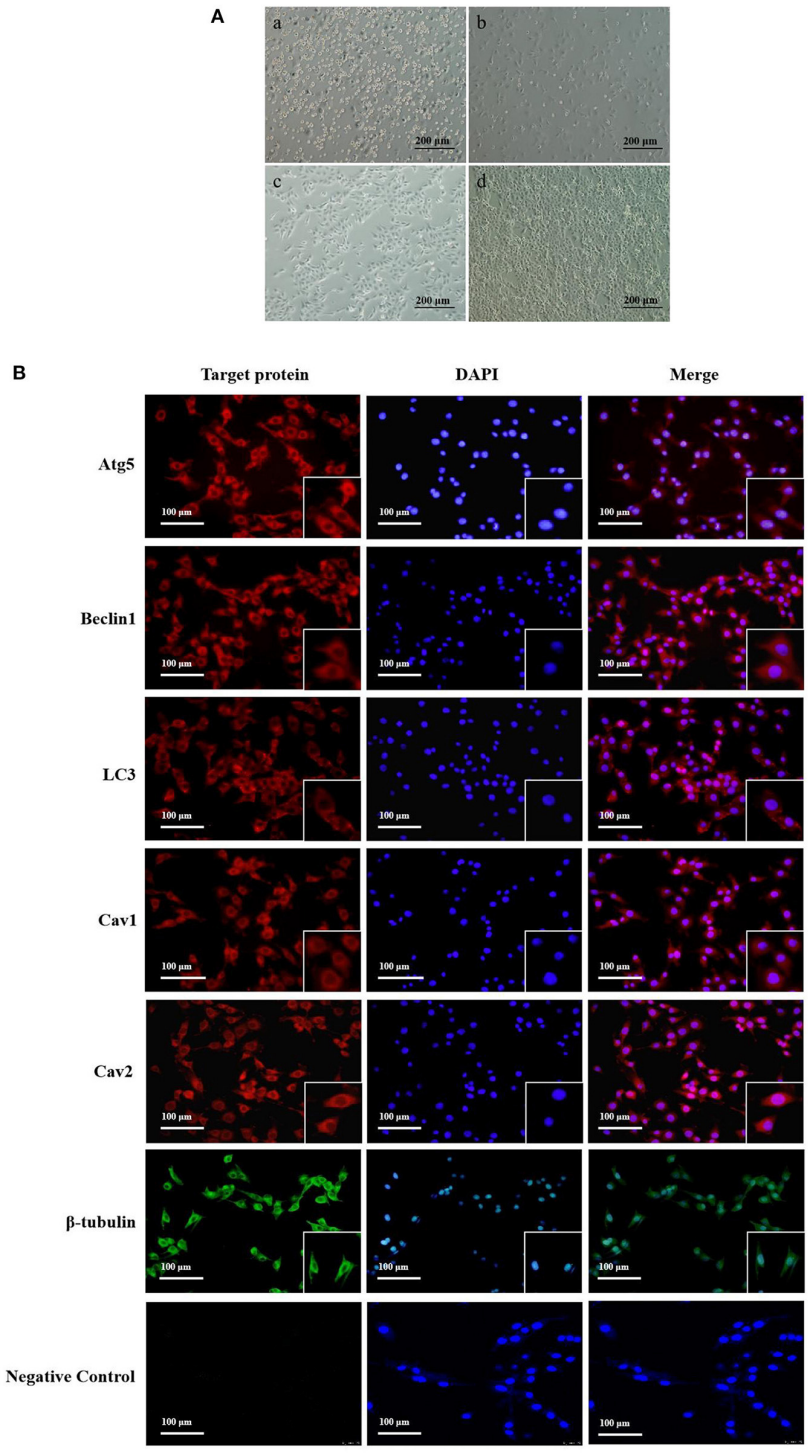
Yak cumulus cells culture and distribution of Atg5, Beclin1, LC3, Cav1, Cav2 protein in CCs. **(A)** The growth pattern of the second generation yak cumulus cells (10×) (a–d showed cell growth at 6, 24, 48, and 72 h, respectively). **(B)** Localization of target proteins in yak cumulus cells. Red fluorescence, Atg5, Beclin1, LC3, Cav1, and Cav2 protein, respectively; Green fluorescence, β-tubulin protein; Blue fluorescence: cell nucleus.

We successfully cultured yak cumulus cells *in vitro* (Figure 1A). At 6 h after cell seeding, we observed that about 10% of the cells had adherent growth, which expanded from round to spindle (Figure 1A-a). After 24 h, 30–40% of the cells adhered to the bottom of the culture flask, and the cells began to gather, with large gaps between the cells (Figure 1A-b). After 48 h, the gap decreased, and the adhesion of cumulus cells reached 60–70%, and some cells were polygonal (Figure 1A-c). After 72 h, a layer of adherent cells of uniform size and good shape was formed at the bottom of the bottle (Figure 1A-d).

Immunofluorescence results showed that autophagy proteins Atg5, Beclin1, and LC3 were all expressed in yak cumulus cells and mainly expressed in the cytoplasm, with feeble nuclear expression. Cav1 and Cav2 proteins were also expressed in normal growth yak cumulus cells and mainly distributed in the cell membrane and cytoplasm. The nuclear expression was weak (Figure 1B).

### 3.2. EGF affects autophagy in yak cumulus cells by regulating the expression of Atg5, Beclin1, and LC3

**Figure 2:**
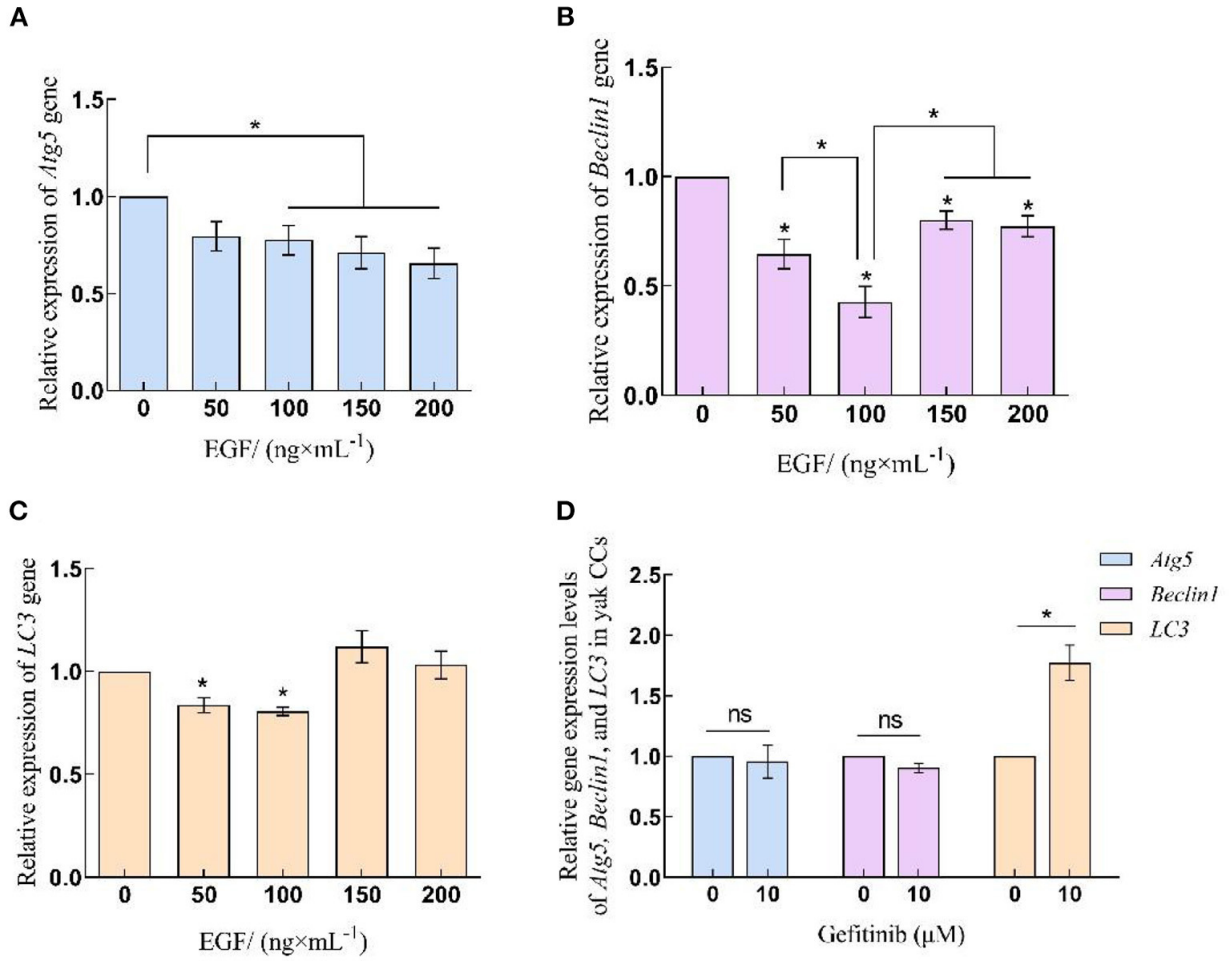
Relative expression levels of Atg5, Beclin1 and LC3 gene in yak CCs treated with different concentrations of EGF and the optimal concentration of gefitinib (EGFR inhibitor). **(A–C)** Relative mRNA expression levels of Atg5, Beclin1, and LC3 in yak CCs (EGF group). **(D)** Relative mRNA expression levels of Atg5, Beclin1, and LC3 in yak CCs (Gefitinib group). *on the bars indicates values that differ significantly (*P < 0.05).

qRT-PCR results showed that the expression of autophagy-related genes Atg5, Beclin1, and LC3 in yak cumulus cells treated with different concentrations of EGF showed some inhibition in a certain concentration range. Among them, the expression of Atg5 in 100–200 ng/mL EGF treatment groups was significantly lower than that of the control group (0 ng/mL) (*P* < 0.05), but there was no significant expression of Atg5 between each treatment group in 100–200 ng/mL (*P* > 0.05). After treatment with different concentrations of EGF, Beclin1, and LC3 gene expression levels declined firstly and then rebounded. The relative expression levels of the Beclin1 gene in each treatment group were significantly lower than those in the control group (*P* < 0.05) and lowest at 100 ng/mL, significantly different from other treatment groups (*P* < 0.05). LC3 gene expression in 50 and 100 ng/mL EGF treatment groups decreased significantly, lower than the control group (0 ng/mL) (*P* < 0.05). After inhibiting EGFR, the relative expression of Atg5 and Beclin1 genes did not change significantly (*P* > 0.05), while LC3 gene expression increased significantly (*P* < 0.05) (Figure 2).

**Figure 3:**
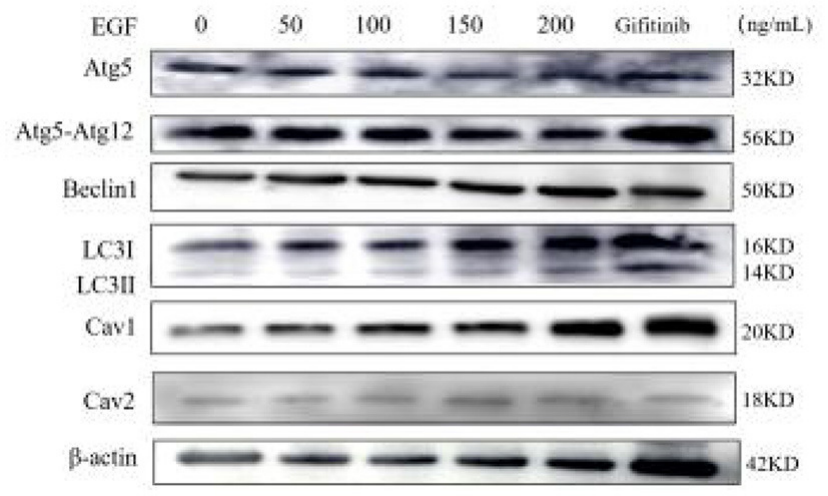
Western-blot detection of Atg5, Atg5-Atg12, Beclin1, LC3I, LC3II, Cav1, Cav2, and β-actin proteins under different EGF concentrations and EGFR inhibitor action group. From left to right: 0, 50, 100, 150, 200 ng/mL EGF group and EGFR inhibitor gefitinib group.

**Figure 4:**
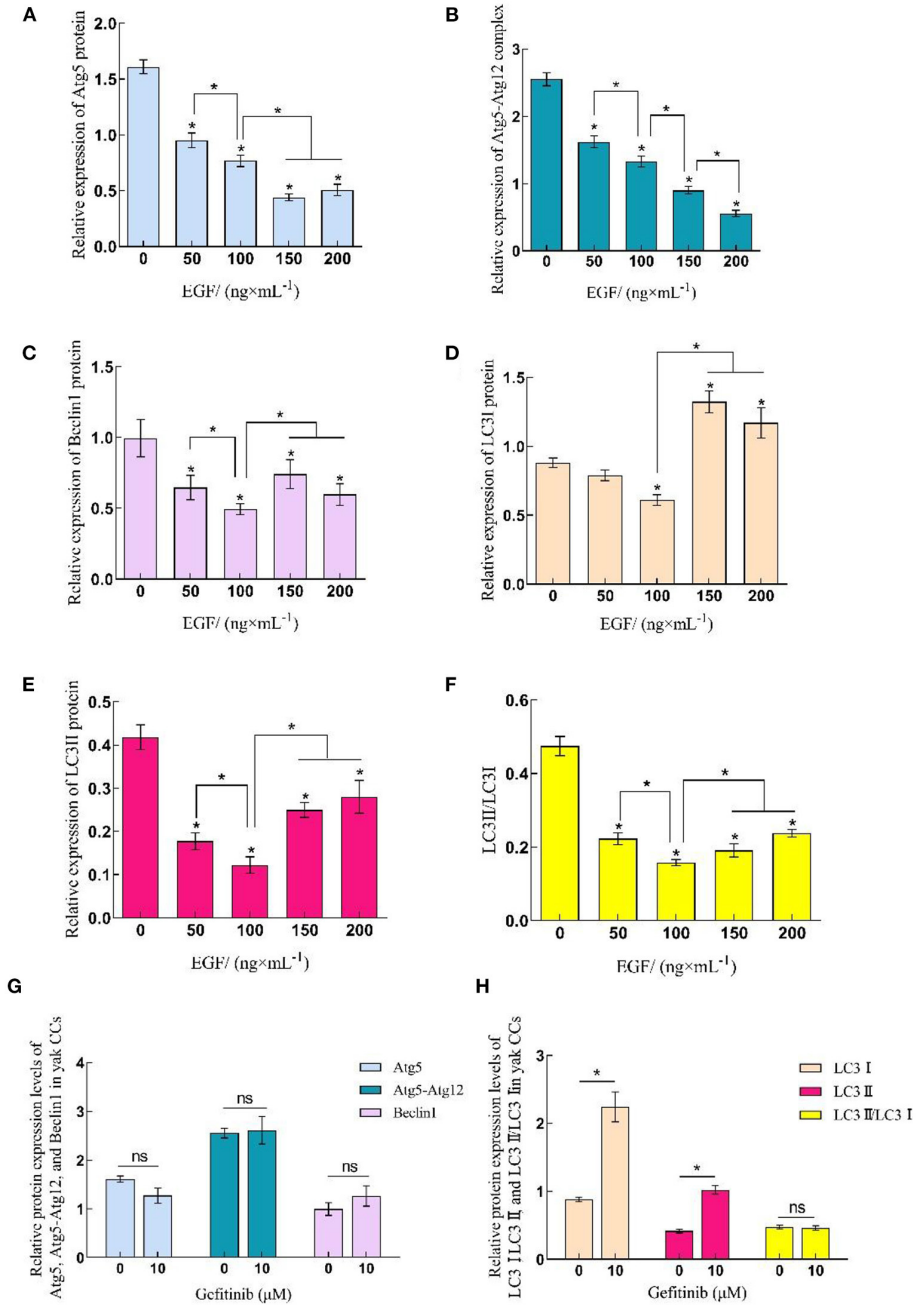
Relative expression levels of Atg5, Beclin1, and LC3 protein in yak CCs treated with different concentrations of EGF and the optimal concentration of gefitinib (EGFR inhibitor). **(A–F)** Relative protein expression levels of Atg5, Atg5-Atg12, Beclin1, LC3I, LC3II, and LC3II/LC3I in yak CCs (EGF group). **(G, H)** Relative protein expression levels of Atg5, Atg5-Atg12, Beclin1, LC3I, LC3II, and LC3II/LC3I in yak CCs (Gefitinib group). *on the bars indicates values that differ significantly (*P < 0.05).

Western blot was used to detect protein samples of cells in EGF treatment groups (Figure 3). The results showed that the expression of autophagy-related proteins in yak cumulus cells was concentration-dependent with EGF, displaying that the expression level of Atg5 and Atg5–Atg12 complex decreased with the increase of EGF concentration and the expression level of each EGF treatment group was significantly lower than the control group (0 ng/mL) (*P* < 0.05), and the expression level of the 200 ng/mL treatment group was the lowest (*P* < 0.05). In addition, the expressions of Beclin1, LC3I, LC3II, and LC3II/LC3I all reached the lowest at 100 ng/mL treatment group (*P* < 0.05) but increased at 150 and 200 ng/mL, and the expression of LC3I protein in the 150 and 200 ng/mL treatment groups increased significantly (*P* < 0.05), which was higher than that in the control group (0 ng/mL). After EGFR inhibiting, the expression levels of Atg5, Atg5–Atg12, and Beclin1 and LC3II/LC3I did not change significantly (*P* > 0.05), while the expression levels of LC3I and LC3II protein both increased significantly (*P* < 0.05) (Figures 3, 4).

### 3.3. EGF affects caveolin-mediated endocytosis in yak cumulus cells by regulating the expression of Cav1 and Cav2

**Figure 5:**
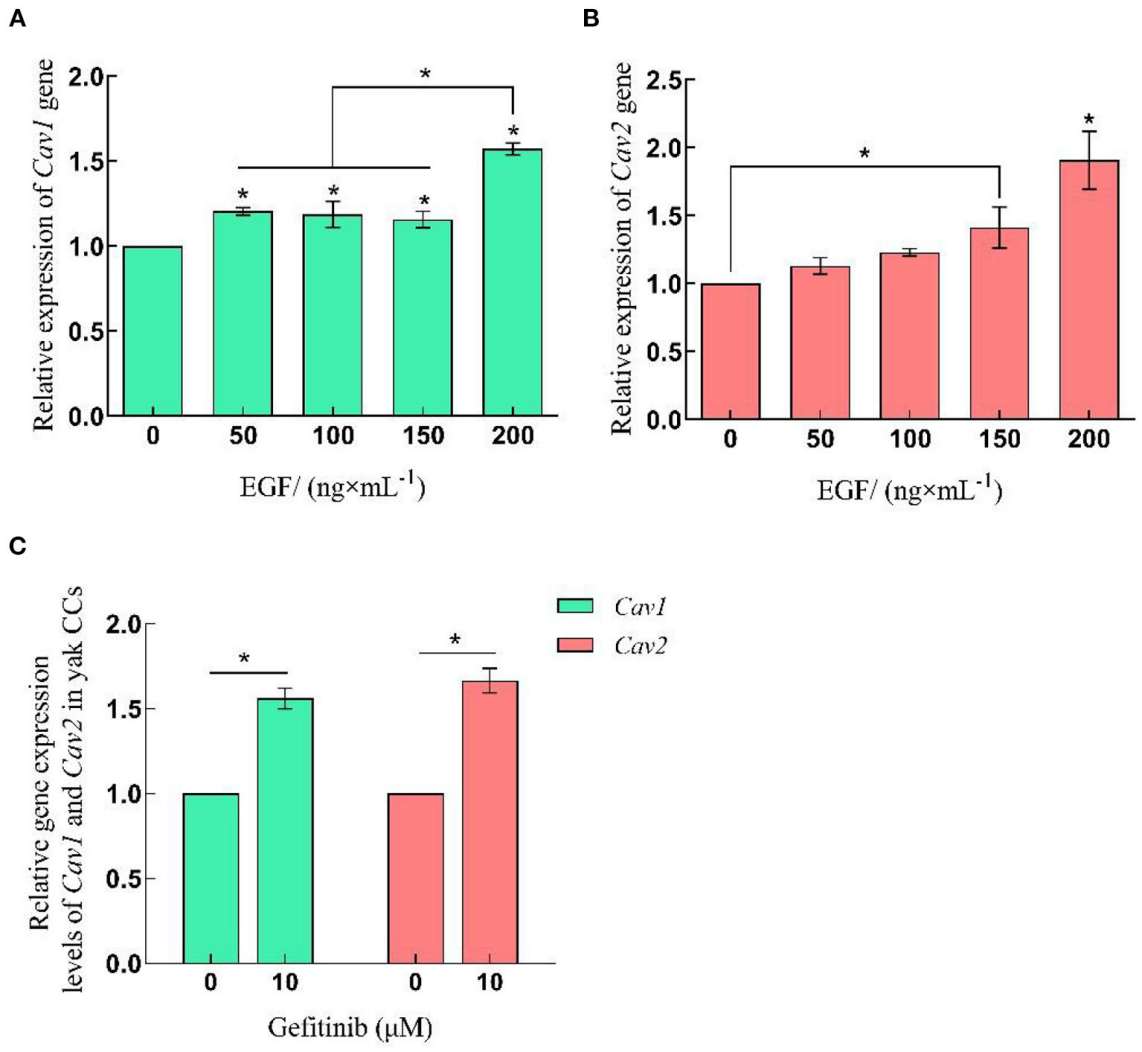
Relative expression levels of Cav1 and Cav2 gene in yak CCs treated with different concentrations of EGF and the optimal concentration of gefitinib (EGFR inhibitor). **(A, B)** Relative mRNA expression levels of Cav1 and Cav2 in yak CCs (EGF group). **(C)** Relative mRNA expression levels of Cav1 and Cav2 in yak CCs (Gefitinib group). *on the bars indicates values that differ significantly (*P < 0.05).

**Figure 6:**
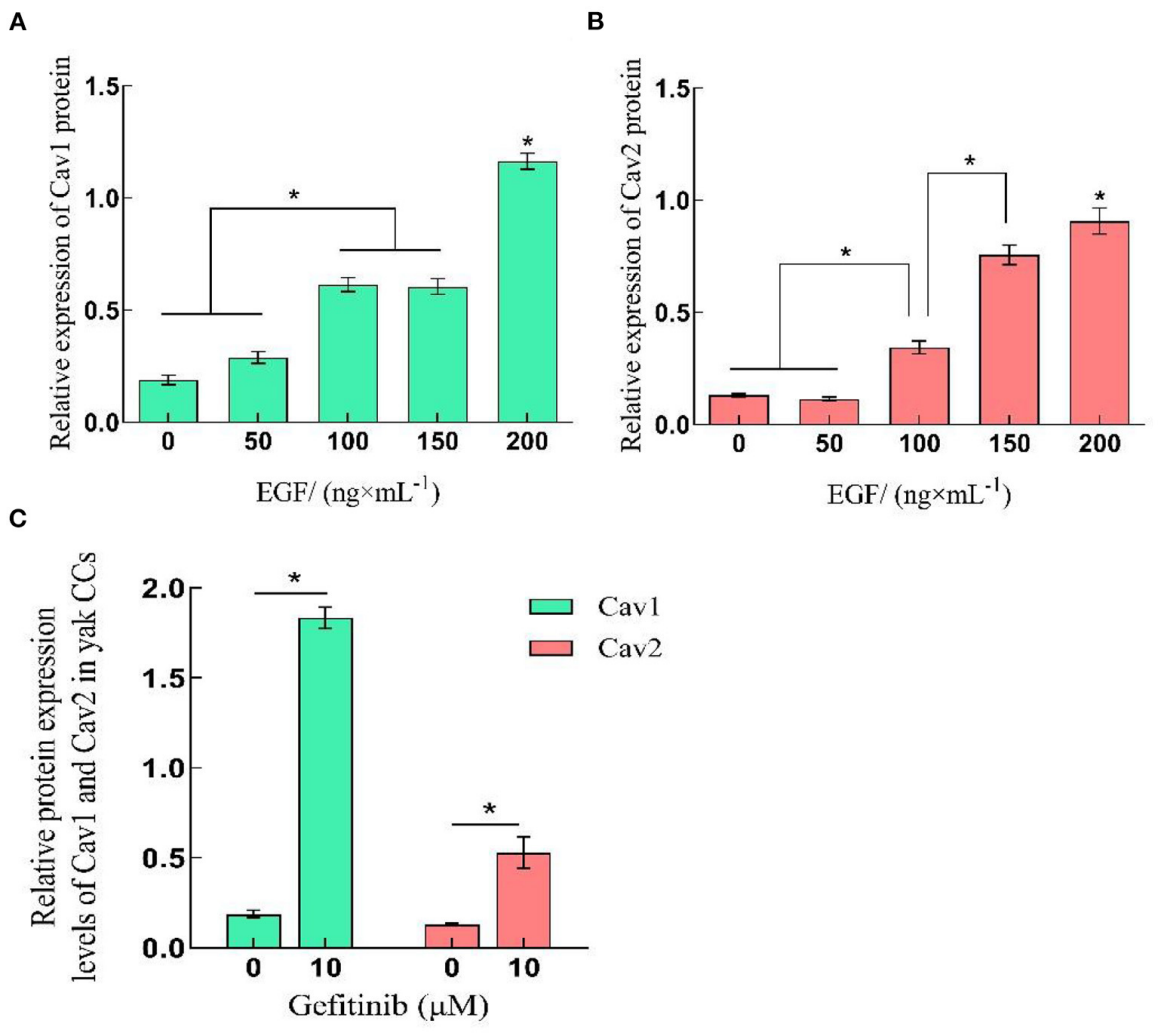
Relative expression levels of Cav1 and Cav2 protein in yak CCs treated with different concentrations of EGF and the optimal concentration of gefitinib (EGFR inhibitor). **(A, B)** Relative protein expression levels of Cav1 and Cav2 in yak CCs (EGF group). **(C)** Relative protein expression levels of Cav1 and Cav2 in yak CCs (Gefitinib group). *on the bars indicates values that differ significantly (*P < 0.05).

The qRT-PCR and Western-blot results showed that the relative expression levels of Cav1 and Cav2 in yak cumulus cells increased with the improvement of EGF concentration, which was the highest at 200 ng/mL, significantly higher than other treatment groups (*P* < 0.05). After EGFR inhibition acted on cells, the relative expression of Cav1 and Cav2 genes and proteins increased, which were significantly higher than that of the control group (0 ng/mL) (*P* < 0.05) (Figures 3, 5, 6).

## 4. Discussion

Autophagy and endocytosis are important ways of cell homeostasis during mammalian follicle and oocyte development. It is essential for follicle cell growth, material transport, oxidative stress regulation, and apoptosis (32). As a typical plateau animal, yak has a unique regulatory mechanism for energy metabolism and oxidative stress regulation. Autophagy and endocytosis can help cells cope with the extreme environment, but their regulatory mechanisms in yak follicle cells are not clear. In this study, we detected the expression of autophagy key proteins Atg5, Beclin1, LC3, and endocytosis proteins Cav1 and Cav2 in untreated yak cumulus cells by indirect immunofluorescence, which were mainly distributed in the cytoplasm of cumulus cells. The protein localization results were consistent with their functional characteristics. These results indicated that these key regulatory molecules could help yak cumulus cells maintain autophagy and endocytic activity during development.

Recently, some studies have pointed out that the addition of melatonin, trehalose and other substances *in vitro* contributes to oocyte development (33, 34), while EGF, as an important paracrine factor in follicles and a commonly used nutritional supplement *in vitro* culture, plays a crucial role in regulating cumulus cell growth, oocyte maturation and early embryonic development (19, 21, 23). To further investigate the function of EGF in the regulation of autophagy and endocytosis of cumulus cells, we detected the expression of related genes and proteins in cumulus cells treated with different concentrations of EGF and gefitinib. It was found that the expression levels of Beclin1, LC3I, LC3II, and LC3II/LC3I in cells treated with different concentrations of EGF were significantly decreased in each treatment group (*P* < 0.05), showed concentration dependence, and the 100 ng/mL treatment group had the lowest expression level (*P* < 0.05). It indicated that 100 ng/mL EGF could effectively inhibit the activation of the autophagy pathway in yak cumulus cells. At this moment, the formation of autophagosome and cell autophagy activation reached the lowest level to help stabilize cells in the face of nutrient deprivation. Studies have shown that autophagy can promote apoptosis in porcine ovarian granulosa cells, and cells with low autophagy level have better growth status (35). Abnormal activation of autophagy in human granulosa cells affects follicle development and causes polycystic ovary synthesis and other diseases (36). This study further indicated that the addition of exogenous EGF could significantly inhibit the autophagy level of yak cumulus cells, which may have a positive effect on oocyte development. It has also been found that 100 ng/mL EGF can significantly improve the development rate of yak embryos *in vitro*. Therefore, 100 ng/mL can be used as the reference concentration of EGF in the co-culture system of yak oocytes and cumulus cells. Notably, LC3I protein and LC3II protein were significantly up-regulated by gefitinib treatment, while the conversion rate of LC3I to LC3II protein remained at the same level, indicating that inhibition of EGFR increased the formation of autophagosomes, and EGFR-mediated classical signaling pathway played a dominant role in the regulation of autophagy activity in yak cumulus cells. In addition, we found that when the doses of EGF were increased to 150 and 200 ng/mL, the inhibitory effects on Beclin1 and LC3 proteins were not significant. The expression level of LC3I protein was even upregulated, indicating that a high dose of EGF may promote autophagy. Studies have shown that EGF acts on cells mainly through internalization after binding to its receptor EGFR. Under the action of EGF, EGFR rapidly leaves the endoplasmic capsule/lipid raft and undergoes signal transduction and endocytosis (37). When EGFR internalizes redundancy, cells can degrade it through the lysosome pathway, making EGFR return to the membrane (38). Therefore, it is speculated that the LC3I protein expression is increased and autophagosomes are generated to degrade excessive internalized EGFR and maintain cell homeostasis when the EGF concentration is higher than 150 ng/mL.

At the same time, we compared the expression levels of Cav1 and Cav2 at the transcriptional and translational levels of different concentrations of EGF. Cav1 and Cav2 proteins are usually co-expressed in cell membranes and participate in endocytosis to realize material exchange. Cav2 must bind to Cav1 to form oligomers and locate in cavities. Under the action of EGF, EGFR rapidly leaves the cytoplasmic membrane for signal transduction and endocytosis (39–41). The gene and protein expressions of Cav1 and Cav2 increased with the increase of EGF concentration in all EGF treatment groups, indicating that EGF can promote the protein expression of Cav1 and Cav2 and participate in the regulation of yak cumulus cell endocytosis. Studies have shown that gefitinib can effectively inhibit the conduction of the EGF signaling pathway, and overexpression of Cav1 in tumor cells can reduce the inhibitory effect of gefitinib (40, 42). Notably, in this study, the expression of Cav1 and Cav2 was also significantly increased in the EGFR-inhibited group compared with the control group (*P* < 0.05), indicating that the EGFR-mediated signaling pathway is not the main pathway regulating the expression of endocytosis-related proteins in yak cumulus cells. We hypothesized that yak cumulus cells may reduce the inhibitory effect of EGFR tyrosine kinase by increasing the expression of Cav1 and helping the normal growth of yak cumulus cells. In addition, Cav1 is involved in the assembly and development of primordial follicles and is an essential regulatory molecule for early ovarian development. It is essential for gonadal development, and the downregulation of its expression can lead to abnormal follicle development. The oocyte syncytium is not separated, and the proliferation and migration ability of the cells are reduced (43, 44). In this study, exogenous EGF was added to increase the expression of Cav1 in cumulus cells of yak, suggesting that the increase of EGF content may increase the expression of Cav1 in cumulus cells, enhance the exchange of cumulus cells, and promote follicle development. The specific mechanism needs to be further studied.

In conclusion, we demonstrated that the addition of exogenous EGF was involved in regulating the expression of Atg5, Beclin1, LC3, Cav1, and Cav2 in cultured yaks cumulus cells *in vitro*, and then affected their autophagy and endocytosis levels. The expression of EGF was dose-dependent. By comparing the data of each group, the optimal inhibitory concentration of EGF on autophagy was considered to be 100 ng/mL. This will be helpful to further explore the regulatory effects of growth factors on mammalian germ cells.

## Data availability statement

The raw data supporting the conclusions of this article will be made available by the authors, without undue reservation.

## Ethics statement

The animal study was reviewed and approved by Animal Ethics Committee of Gansu Agricultural University.

## Author contributions

Data curation: RM and JW. Formal analysis: MW. Funding acquisition: SY. Investigation: LW. Methodology: YP, RM, JW, LZ, and HZ. Supervision: YC and SY. Writing—original draft: RM and YP. Writing—review and editing: YP, YC, and SY. All authors contributed to the article and approved the submitted version.
